# Long‐term follow‐up results from KEYNOTE‐041: Phase 1b study of pembrolizumab in Japanese patients with advanced melanoma

**DOI:** 10.1111/1346-8138.17002

**Published:** 2024-03-26

**Authors:** Kenji Yokota, Tatsuya Takenouchi, Yasuhiro Fujisawa, Satoshi Fukushima, Hiroshi Uchi, Takashi Inozume, Yoshio Kiyohara, Hisashi Uhara, Kazuhiko Nakagawa, Hiroshi Furukawa, Shirong Han, Masaru Watanabe, Kazuo Noguchi, Naoya Yamazaki

**Affiliations:** ^1^ Department of Dermatology Nagoya University Graduate School of Medicine Nagoya Japan; ^2^ Department of Dermatology Niigata Cancer Center Hospital Niigata Japan; ^3^ Department of Dermatology, Faculty of Medicine Ehime University Ehime Japan; ^4^ Department of Dermatology and Plastic Surgery, Faculty of Life Sciences Kumamoto University Kumamoto Japan; ^5^ Department of Dermatologic Oncology, National Hospital Organization Kyusyu Cancer Center Fukuoka Japan; ^6^ Chiba University Chiba Japan; ^7^ Division of Dermatology Shizuoka Cancer Center Hospital Shizuoka Japan; ^8^ Department of Dermatology Sapporo Medical University, School of Medicine Sapporo Hokkaido Japan; ^9^ Department of Medical Oncology, Faculty of Medicine Kindai University Osaka Japan; ^10^ Department of Plastic and Reconstructive Surgery Aichi Medical University Hospital Nagakute Aichi Japan; ^11^ MSD K.K Tokyo Japan; ^12^ Department of Dermatologic Oncology National Cancer Center Hospital Tokyo Japan

**Keywords:** advanced melanoma, Japanese, long‐term follow‐up, pembrolizumab, subtype

## Abstract

Pembrolizumab demonstrated an acceptable safety profile and promising antitumor activity in Japanese patients with advanced melanoma in the phase 1b KEYNOTE‐041 (Study of Pembrolizumab [MK‐3475] in Participants With Advanced Melanoma) trial. To evaluate the long‐term efficacy and safety of pembrolizumab in Japanese patients with advanced melanoma in KEYNOTE‐041. The current analysis reports results of additional follow‐up of approximately 12 months since the initial analysis. Eligible patients had locally advanced (unresectable stage III) or metastatic (stage IV) melanoma not amenable to local therapy and had received two or fewer prior systemic therapies. Pembrolizumab 2 mg/kg was given every 3 weeks for up to 2 years or until confirmed progression or unacceptable toxicity. Primary end points included safety, tolerability, and overall response rate (ORR) per Response Evaluation Criteria in Solid Tumors version 1.1 by independent central review. The data cutoff for this analysis was August 30, 2017. Forty‐two patients were followed up for a median of 22.3 months (range, 2.63–30.82 months). The ORR was 24.3% (nine of 37 evaluable patients [95% confidence interval (CI), 11.8%–41.2%]). Two patients with partial response at the time of the initial analysis achieved complete response. The median overall survival (OS) was 25.1 months (95% CI, 13.1–not reached] and the 30‐month OS rate was 46.3% (95% CI, 29.8%–61.3%). The median duration of response was not reached. Treatment‐related adverse events (TRAEs) were reported in 78.6% of patients; the incidence of grade 3 to 5 TRAEs was 23.8%. No additional treatment‐related deaths occurred since the initial analysis. Pembrolizumab provided durable antitumor activity and an acceptable safety profile in Japanese patients with advanced melanoma.

## 1 INTRODUCTION

The recent development of immune checkpoint inhibitors has dramatically improved outcomes for patients with melanoma; however, access to these agents varies significantly by country.^1^, ^2^ Although melanoma is relatively rare in Japan, immune checkpoint inhibitors, including pembrolizumab, nivolumab, and ipilimumab, are available and constitute a key part of the therapeutic landscape for melanoma.^3^ Pembrolizumab is a highly selective monoclonal antibody that blocks the interaction between PD‐1 (programmed cell death 1 protein) and its ligands PD‐L1 (programmed cell death ligand 1) and PD‐L2, thereby promoting cytotoxic T‐cell–mediated antitumor responses.^4^ Pembrolizumab has demonstrated durable antitumor activity in melanoma and is well tolerated.^5^, ^6^, ^7^, ^8^

KEYNOTE‐041 (Study of Pembrolizumab [MK‐3475] in Participants With Advanced Melanoma) (ClinicalTrials.gov identifier: NCT02180061) is a single‐arm phase Ib study investigating pembrolizumab monotherapy in Japanese patients with unresectable or metastatic melanoma.^9^ The results of the initial analysis of KEYNOTE‐041 with a median follow‐up of 10.3 months showed that pembrolizumab had promising antitumor activity.^9^ The confirmed overall response rate (ORR) was 24.1% (95% confidence interval [CI], 10.3–43.5) for patients with cutaneous melanoma and 25.0% (95% CI, 3.2–65.1) for patients with mucosal melanoma. The median duration of response (DOR) was not reached (NR) in either population. The 12‐month overall survival (OS) rate was 82.7% for patients with cutaneous melanoma and 51.4% for patients with mucosal melanoma. The safety profile of pembrolizumab in Japanese patients was similar to that reported in previous clinical studies of predominately white populations.^9^, ^10^, ^11^, ^12^ Thirty‐four patients (81.0%) experienced at least one treatment‐related adverse event (TRAE), the most common of which were pruritus, maculopapular rash, malaise, and hypothyroidism.^9^ Grade 3 to 5 TRAEs occurred in eight patients (19.0%), with anemia as the only event reported in at least two patients. Two treatment‐related deaths occurred: one from an unknown cause and one due to cerebral hemorrhage. These results support pembrolizumab as an effective and well‐tolerated treatment option in Japanese patients with advanced melanoma.

The PD‐1 inhibitor nivolumab has been shown to produce durable survival benefit in patients with melanoma, including Japanese patients.^13^, ^14^, ^15^, ^16^ In an open‐label, phase 2 study of Japanese patients with previously untreated advanced or recurrent melanoma, the median OS with nivolumab was 32.9 months and the 5‐year OS rate was 26.1%.^16^ Nivolumab and ipilimumab combination therapy has also been associated with a long‐term survival benefit in Japanese patients.^17^, ^18^ In clinical practice of Japanese patients with previously untreated advanced or recurrent melanoma, the median PFS and OS with nivolumab and ipilimumab were 6.5 months and 25.3 months, respectively.^18^

Here, we report the final analysis of KEYNOTE‐041, providing long‐term safety and efficacy outcomes after a median follow‐up of 22.3 months. In addition, we describe outcomes in patient subgroups by cutaneous melanoma subtype.

## 2 MATERIALS AND METHODS

### 2.1 Patients

As previously described,^9^ eligible patients were 20 years or older with locally advanced (unresectable stage III) or metastatic (stage IV) melanoma not amenable to local therapy; had received up to two prior lines of therapy (excluding adjuvant or neoadjuvant therapy); had at least one measurable lesion per Response Evaluation Criteria in Solid Tumors version 1.1 (RECIST 1.1); had an Eastern Cooperative Oncology Group (ECOG) performance status of 0 or 1; and had adequate hematologic, hepatic, and renal functions. Patients were excluded if they had received chemotherapy, radiotherapy, or biological therapy within 4 weeks of first dose of trial treatment; had received prior treatment with a PD‐1, PD‐L1, or CTLA‐4 (cytotoxic T‐lymphocyte–associated protein 4) inhibitor; had untreated and/or unstable central nervous system metastasis; or had an active autoimmune disease.

Patients provided written informed consent. The protocol was approved by the institutional review boards or independent ethics committees of the participating institutions, and the trial was conducted in accordance with Good Clinical Practice guidelines and the Declaration of Helsinki.

### 2.2 Study design

KEYNOTE‐041 is an open‐label, nonrandomized, multicenter, phase 1b trial. The detailed methodology and initial analysis of this trial have been previously published.^9^ In brief, patients received pembrolizumab 2 mg/kg once every 3 weeks administered intravenously over a 30‐min period. Treatment continued until disease progression, unacceptable toxicity, investigator's decision to discontinue treatment, withdrawal of patient consent, or until the patients had received 24 months of treatment. Patients with a confirmed complete response (CR) who had received pembrolizumab for at least 6 months were allowed to discontinue therapy after receiving at least two doses after the determination of a CR.

### 2.3 End points

The primary objectives of KEYNOTE‐041 were to determine the safety and tolerability of pembrolizumab and to evaluate ORR per RECIST 1.1 as assessed by independent central review in patients with advanced cutaneous melanoma. Secondary objectives included evaluating DOR and progression‐free survival (PFS) per RECIST 1.1 and OS in patients with advanced cutaneous melanoma. Exploratory objectives included evaluating ORR, DOR, and PFS per RECIST 1.1, and OS in patients with advanced mucosal melanoma.

### 2.4 Assessments

Radiographic imaging was performed every 6 weeks and tumor response was assessed per RECIST 1.1 by central review. If clinically stable, patients with first radiologic evidence of disease progression per RECIST 1.1 were permitted per investigator decision to continue receiving pembrolizumab until a second scan performed at least 4 weeks later confirmed progression. Safety was monitored throughout the study, and all adverse events (AEs) were graded per the National Cancer Institute Common Terminology Criteria for Adverse Events version 4.0.

As previously described,^9^ PD‐L1 expression was assessed in formalin‐fixed tumor samples (from an archival tissue sample or a newly obtained biopsy of a tumor lesion not previously irradiated) using a PD‐L1 IHC 22C3 pharmDx assay (Agilent). PD‐L1 status was determined at a central laboratory. PD‐L1 positivity was defined as membranous PD‐L1 expression in ≥1% of tumor cells and associated immune cells. Both PD‐L1–positive and PD‐L1–negative patients were enrolled in the trial as predefined subgroups.

### 2.5 Statistical analysis

This was the final analysis of the KEYNOTE‐041 study (data cutoff: August 30, 2017). Study follow‐up was defined as time from randomization to death or database cutoff if the patient was alive. The efficacy population included all allocated patients with measurable disease, those with a baseline scan and a post baseline scan, or those who discontinued the trial because of progressive disease (PD) or TRAE. The safety population included all allocated patients who received at least one dose of study treatment. The primary end point for efficacy (confirmed ORR per RECIST 1.1 by central review) was assessed in the cutaneous melanoma population. For the ORR, the 95% CI and one‐sided *p* value for testing the null hypothesis (ORR, 10%) were calculated based on a binomial distribution. With approximately 28 evaluable patients with advanced cutaneous melanoma, the study had an approximately 90% power to detect a 25% difference in ORR with a type I error rate of 2.5% if the true ORR was 35%. PFS and OS were estimated using the Kaplan–Meier method, with censoring of data for patients alive or lost to follow‐up at the time of last contact.

## 3 RESULTS

### 3.1 Patients and treatment

KEYNOTE‐041 enrolled 42 patients from 12 sites; all patients were treated with pembrolizumab. At data cutoff (August 30, 2017), median follow‐up was 22.3 months (range, 2.63–30.82 months) and 33 patients had discontinued study treatment. The most common reason for treatment discontinuation was PD (*n* = 20; 47.6%) followed by an AE (*n* = 6; 14.3%), clinical progression (*n* = 6; 14.3%), and physician decision (*n* = 1; 2.4%). Median treatment duration was 7.62 months (range, 0.03–23.82 months). Seventeen patients (40.5%) went on to receive one or more subsequent oncologic treatments, including immunotherapies (anti–CTLA‐4, *n* = 8; anti–PD‐1, *n* = 6), targeted therapies (BRAF inhibitor, *n* = 4; BRAF and MEK inhibitor, *n* = 1), chemotherapy (*n* = 3), and other (*n* = 1). All subsequent treatments were given after discontinuation of pembrolizumab. The median time to subsequent systemic therapy was 10.58 months (range, 1.18–27.27 months). Patient demographic and baseline clinical characteristics are provided in Table 1. Most patients were men (61.9%), had an ECOG performance status of 0 (81.0%), had *BRAF* wild‐type disease (78.6%), had a normal level of lactate dehydrogenase (97.6%), and had received no prior systemic therapy for advanced melanoma (59.5%). The majority of patients had cutaneous melanoma (81.0%), of which the most common subtypes were acral lentiginous melanoma (28.6%) and nodular melanoma (23.8%).

**TABLE 1:** Baseline characteristics.

Characteristic	All treated patients (*N* = 42)
Sex, *n* (%)	
Men	26 (61.9)
Women	16 (38.1)
ECOG performance scale, *n* (%)	
0	34 (81.0)
1	8 (19.0)
Tumor types, *n* (%)	
Cutaneous melanoma	34 (81.0)
Nodular melanoma	10 (23.8)
Superficial spreading melanoma	7 (16.7)
Lentigo maligna melanoma	1 (2.4)
Acral lentiginous melanoma	12 (28.6)
Not classified	4 (9.5)
Mucosal melanoma	8 (19.0)
*BRAF* status, *n* (%)	
Mutant	7 (16.7)
Wild‐type	33 (78.6)
Undetermined	2 (4.8)
LDH, *n* (%)	
Normal	41 (97.6)
Elevated	1 (2.4)
PD‐L1 expression^a^	
Positive	21 (50.0)
Negative	13 (31.0)
Undetermined	8 (19.0)
Prior systemic therapies, *n* (%)	
None^b^	10 (23.8)
0	15 (35.7)
1	13 (31.0)
2	4 (9.5)
Prior neoadjuvant/adjuvant therapies, *n* (%)	
Yes	25 (59.5)
No	17 (40.5)

Abbreviations: ECOG, Eastern Cooperative Oncology Group; LDH, lactate dehydrogenase.

^a^ Defined as membranous programmed cell death ligand 1 (PD‐L1) expression in >1% of tumor cells and associated immune cells as assessed by immunohistochemistry using the 22C3 antibody.

^b^ No neoadjuvant/adjuvant systemic therapy.

### 3.2 Objective response

Of the 42 patients enrolled, five were excluded from the efficacy analyses because of the absence of measurable lesions at baseline according to central review. Thirty‐seven patients were therefore evaluable for tumor response: 29 had cutaneous melanoma and eight had mucosal melanoma. Of the 37 patients evaluable for response, four achieved CR and five achieved partial response (PR), for an ORR of 24.3% (95% CI, 11.8–41.2) (Table 2). Two patients who had PR at the time of the initial analysis had achieved a CR at the time of the final analysis. Eighteen patients had disease control (CR, PR, or stable disease [SD]) for a disease control rate of 48.6% (95% CI, 31.9–65.6) (Table 2).

**TABLE 2:** Tumor response per RECIST 1.1 by central review by melanoma histology.

	Cutaneous, *n* = 29	Mucosal, *n* = 8	Total, *N* = 37
Overall objective response, *n* (%, 95% CI)	7 (24.1, 10.3–43.5)	2 (25.0, 3.2–65.1)	9 (24.3, 11.8–41.2)
Disease control rate, *n* (%, 95% CI)	14 (48.2, 29.4–67.5)	4 (50.0, 15.7–84.3)	18 (48.6, 31.9–65.6)
Best overall response, *n* (%, 95% CI)			
Complete response	4 (13.8, 3.9–31.7)	0 (0, 0.0–36.9)	4 (10.8, 3.0–25.4)
Partial response	3 (10.3, 2.2–27.4)	2 (25.0, 3.2–65.1)	5 (13.5, 4.5–28.8)
Stable disease	7 (24.1, 10.3–43.5)	2 (25.0, 3.2–65.1)	9 (24.3, 11.8–41.2)
Progressive disease	14 (48.3, 29.4–67.5)	4 (50.0, (15.7–84.3)	18 (48.6, 31.9–65.6)
Nonevaluate	1 (3.4, 0.1–17.8)	0 (0, 0–36.9)	1 (2.7, 0.1–14.2)
Median time to response, (range), months	2.8 (3–4)	4.1 (3–6)	2.8 (3–6)
Median DOR (range), months	NR (4–25+)	NR (8–20+)	NR (4–25+)

Abbreviations: CI, confidence interval; DOR, duration of response; NR, not reached; RECIST 1.1, Response Evaluation Criteria in Solid Tumors version 1.1.

+—Indicates there was no progressive disease by the time of last assessment.

The ORR was 24.1% for patients with cutaneous melanoma (95% CI, 10.3–43.5; seven of 29 patients) and 25.0% for patients with mucosal melanoma (95% CI, 3.2–65.1; two of eight patients). The ORR was 28.6% (95% CI, 11.3–52.2; six of 21 patients) for patients with treatment‐naive disease and 18.8% (95% CI, 4.0–45.6; three 16 patients) for patients who had received one or more prior lines of therapy. The ORR by melanoma location and subtype is presented in Table S1.

### 3.3 OS and PFS

**FIGURE 1:**
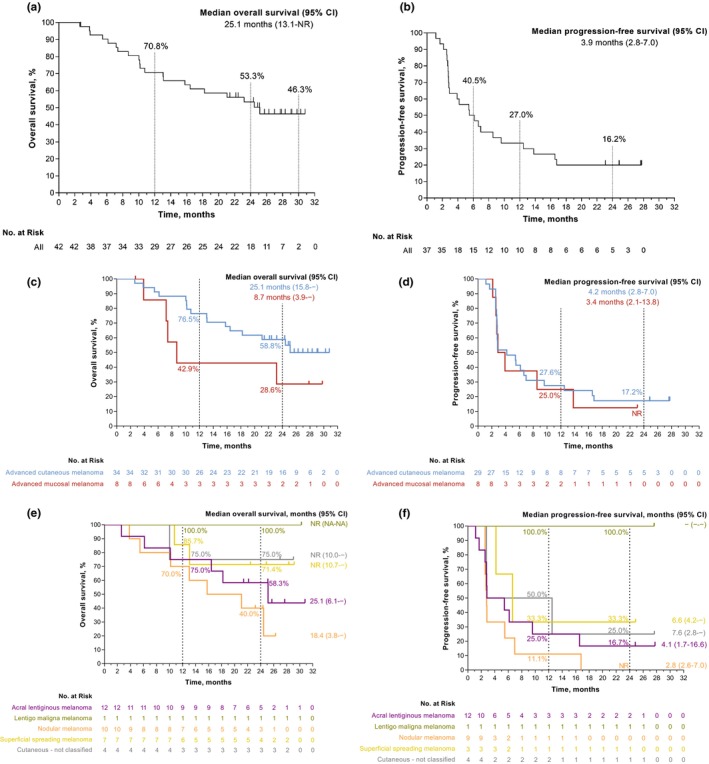
Survival in KEYNOTE‐041 (Study of Pembrolizumab [MK‐3475] in Participants With Advanced Melanoma). (a) Overall survival in the total population. (b) progression‐free survival in the total population. (c) Overall survival by melanoma location. (d) Progression‐free survival by melanoma location. (e) Overall survival by cutaneous melanoma subtype. (f) Progression‐free survival by cutaneous melanoma subtype. CI, confidence interval; NA, not applicable; NR, not reached; − indicates not available based on the data.

At the time of data cutoff, 21 patients had died. The median OS in the total population was 25.1 months (95% CI, 13.1–NR). Kaplan–Meier estimates of OS were 70.8% (95% CI, 54.3–82.2) at 12 months, 53.3% (95% CI, 37.0–67.2) at 24 months, and 46.3% (95% CI, 29.8–61.3) at 30 months (Figure 1a). The median PFS in the total population was 3.9 months (95% CI, 2.8–7.0 months). Kaplan–Meier estimates of PFS were 40.5% (95% CI, 24.9–55.7) at 6 months, 27.0% (95% CI, 14.1–41.8) at 12 months, and 16.2% (95% CI, 6.6–29.6) at 24 months (Figure 1b). Kaplan–Meier estimates of OS and PFS in cutaneous and mucosal melanoma and cutaneous melanoma subtypes are presented in Figures 1c,d and 1e,f respectively. The median PFS and OS by melanoma location and subtype are presented in Table S1.

### 3.4 Treatment duration and time to response

**FIGURE 2:**
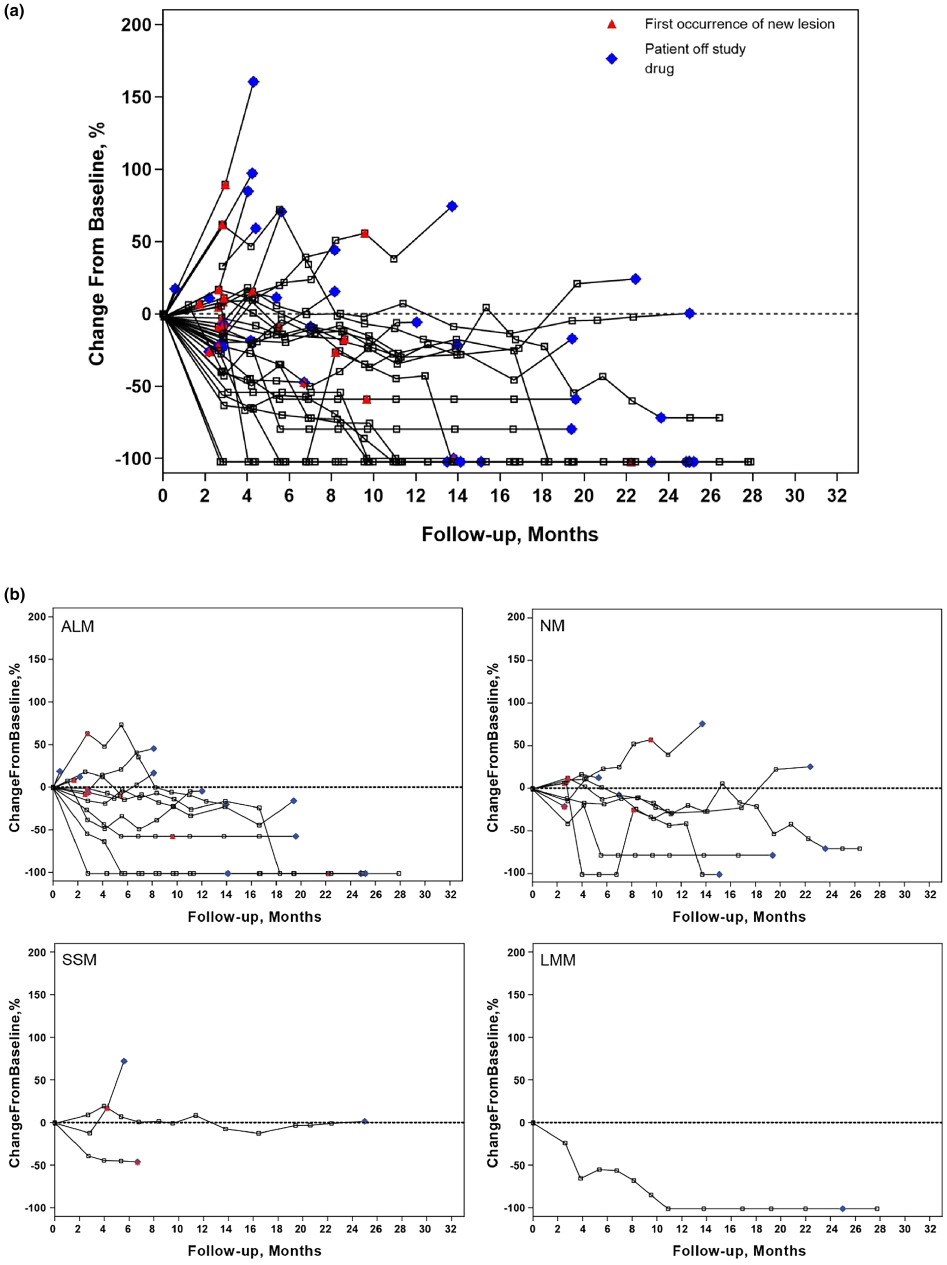
Tumor response dynamics in KEYNOTE‐041 (Study of Pembrolizumab [MK‐3475] in Participants With Advanced Melanoma). (a) Percent change in the sum of the longest diameters of the target lesion from baseline in each patient. (b) Percent change in the sum of the longest diameters of the target lesion from baseline in each patient, shown by cutaneous melanoma subtype. ALM, acral lentiginous melanoma; LMM, lentigo maligna melanoma; NM, nodular melanoma; SSM, superficial spreading melanoma.

The median time to response for patients with cutaneous melanoma was 2.8 months (range, 3–4 months) (Table 2; Table S1 by cutaneous subtype). The median time to response for patients with mucosal melanoma was 4.1 months (range, 3–6 months). The median DOR for both subtypes was NR (cutaneous melanoma: range, 4–25+ months; mucosal melanoma: range, 8–20+ months). The tumor response dynamics for the total population are presented in Figure 2a and by cutaneous melanoma subtype in Figure 2b. Most patients exhibited a sustained reduction in target lesion size over time (Figure 2a). The median time to response and DOR of each tumor type are presented in Table S1.

**FIGURE 3:**
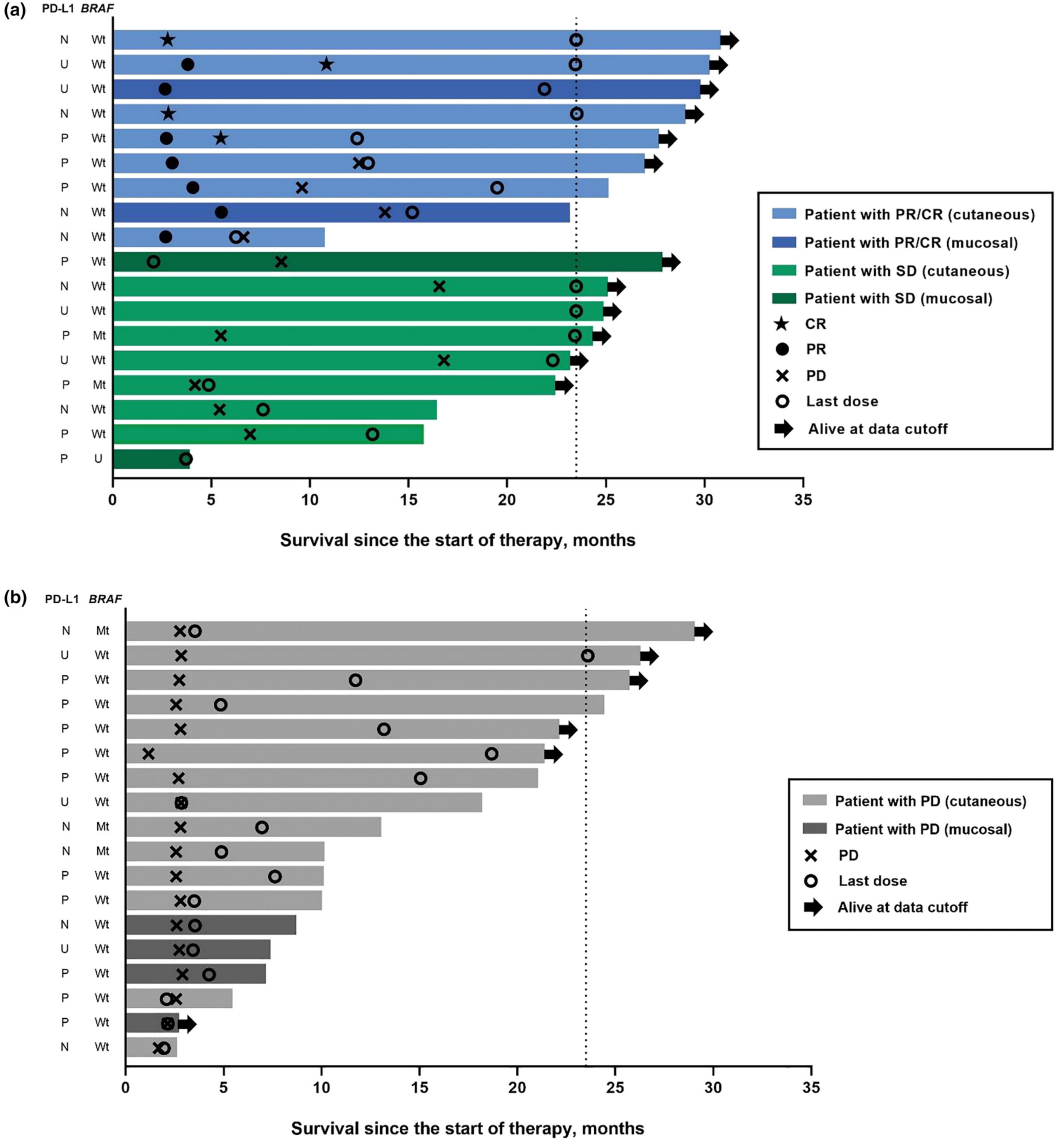
Survival and response duration in (a) patients with complete response (CR), partial response (PR), and stable disease (SD) (b) with progressive disease (PD). Mt, mutant; N, negative; P, positive; U, undetermined; Wt, wild‐type.

Among the nine patients who had CR or PR, the median duration of treatment was 19.5 months (range, 6.2–23.5 months); three of these patients had completed 35 cycles (2 years) of treatment at the time of data cutoff (Figure 3a). At data cutoff, five patients were progression‐free after discontinuation of pembrolizumab treatment. All patients with CR or PR had *BRAF* wild‐type disease. Among the nine patients who had SD, the median duration of treatment was 13.2 months (range, 2.1–23.5 months). Among the 18 patients who had PD, the median duration of treatment was 4.53 months (range, 1.94–23.52 months); one of these patients had completed 35 cycles (2 years) of treatment at the time of data cutoff (Figure 3b). There was no clear trend in response by PD‐L1 status.

### 3.5 Safety

TRAEs of any grade occurred in 78.6% of patients (*n* = 33) (Table 3). The most common TRAEs were pruritus (*n* = 7), rash maculopapular (*n* = 6), and malaise (*n* = 5). TRAEs of grade 3 to 5 occurred in 10 patients (23.8%). Six patients (14.3%) discontinued treatment because of TRAEs. As previously reported, two deaths occurred that were considered related to treatment: one cerebral hemorrhage and one death of unknown cause (fall) (Table 3). Immune‐mediated AEs and infusion reactions of any grade, and regardless of relationship to treatment as assessed by the investigator, occurred in 17 patients (40.5%). Immune‐mediated AEs occurring in ≥5% of patients were hypothyroidism (*n* = 6; 14.3%), severe skin reaction (*n* = 3; 7.1%), and hypophysitis (*n* = 3; 7.1%). Grade 3 to 5 immune‐mediated AEs occurred in five patients (11.9%; grade 3 hypophysitis, colitis, and grade 4 type 1 diabetes in one patient each. Grade 3 severe skin reaction in two patients). Of these four AEs, the severe skin reaction and type 1 diabetes had not occurred at the time of the initial analysis.

**TABLE 3:** Safety summary.

No. of patients (%)	All patients as treated, *N* = 42
Treatment‐related AEs	
Any grade	33 (78.6)
Grade 3–5	10 (23.8)
Led to discontinuation	6 (14.3)
Led to death	2 (4.8)

Treatment‐related AEs	Any Grade, ≥5%	Grade 3–5, ≥0%
Lymphopenia	1 (2.4)	1 (2.4)
Hypophysitis	2 (4.8)	1 (2.4)
Hypothyroidism	4 (9.5)	0
Abdominal pain upper	3 (7.1)	0
Colitis	2 (4.8)	1 (2.4)
Diarrhea	4 (9.5)	0
Cholangitis	1 (2.4)	1 (2.4)
Death of unknown cause (fall)	1 (2.4)^a^	1 (2.4)^a^
Malaise	5 (11.9)	0
Aspartate aminotransferase increased	3 (7.1)	0
Eosinophil count increased	3 (7.1)	0
Hyperglycemia	1 (2.4)	1 (2.4)
Type 1 diabetes	1 (2.4)	1 (2.4)
Encephalopathy	1 (2.4)	1 (2.4)
Cerebral hemorrhage	1 (2.4)^a^	1 (2.4)^a^
Pruritus	7 (16.3)	0
Pemphigoid	1 (2.4)	1 (2.4)
Rash	2 (4.8)	1 (2.4)
Rash maculopapular	6 (14.3)	0
Urticaria	4 (9.5)	0
Vitiligo	3 (7.1)	0

AEs with possible immune cause and infusion reactions	Any grade, ≥0%	Grade 3–5, ≥0%
Colitis	2 (4.8)	1 (2.4)
Hyperthyroidism	2 (4.8)	0
Hypophysitis	3 (7.1)	1 (2.4)
Hypothyroidism	6 (14.3)	0
Infusion reactions	2 (4.8)	0
Pneumonitis	1 (2.4)	0
Severe skin reactions	3 (7.1)	2 (4.8)
Type 1 diabetes	1 (2.4)	1 (2.4)
Uveitis	1 (2.4)	0

Abbreviation: AE, adverse event.

^a^ Grade 5.

## 4 DISCUSSION

Results of the protocol‐specified primary analysis of KEYNOTE‐041 showed that pembrolizumab provided promising antitumor activity in Japanese patients with advanced melanoma and had a safety profile similar to that observed in non‐Japanese patients.^9^ With an additional 12 months of follow‐up in this analysis, pembrolizumab continued to provide antitumor activity and acceptable safety in Japanese patients with advanced melanoma. Responses were durable and patients generally exhibited a sustained reduction in target lesion size over time.

The ORR and median OS results reported in this analysis were lower and shorter than those observed in patients who received pembrolizumab in the phase 3 KEYNOTE‐006 study (ORR, 24% and 33%; median OS: 25.1 months and 32.7 months, respectively).^7^ A possible reason for this is the difference in baseline characteristics between the studies. Almost half of the patients in KEYNOTE‐041 had either acral lentiginous melanoma or mucosal melanoma, which are subtypes generally associated with poor outcome.^19^, ^20^ Although melanoma subtype was not reported in KEYNOTE‐006, previous studies have shown that the incidence of acral lentiginous and mucosal melanoma is higher in Japanese patients than in Western populations.^19^ It is therefore likely that KEYNOTE‐006 included fewer patients with these subtypes, which may have contributed to the reduced benefit seen in the current analysis. Acral and mucosal melanomas are also less likely to harbor *BRAF* mutations than cutaneous melanoma.^21^, ^22^ This may account for why fewer patients in KEYNOTE‐041 had *BRAF* mutations compared with KEYNOTE‐006 (16.7% and 38.5%, respectively). However, there is no clear evidence indicating that *BRAF* status impacts outcome in advanced melanoma treated with pembrolizumab, and patients with both *BRAF* wild‐type and *BRAF* mutant melanoma have been shown to benefit from treatment.^23^ In addition, acral and mucosal melanomas are known to have lower mutational burden than cutaneous melanomas, which may be associated with poor response to checkpoint inhibitors.^24^, ^25^ In a phase 2 trial that investigated nivolumab monotherapy in Japanese patients with melanoma, a high proportion of patients had acral lentiginous (29.2%) and mucosal melanoma (25.0%), and the ORR and 5‐year OS rates (34.8% and 26.1%, respectively) were lower when compared with those in studies of predominantly Western populations such as CheckMate 067 (ORR, 45%; 5‐year OS, 44%).^15^, ^16^, ^26^ These differences in patient characteristics may therefore have contributed to the lower ORR and shorter median OS observed in the current study compared with historical trials.^7^ It should also be noted that the number of patients included in KEYNOTE‐041 was relatively small compared with historical trials such as KEYNOTE‐006, which limits definitive conclusions.

Pembrolizumab has previously demonstrated sustained disease control over a long period of time in patients with advanced melanoma.^7^ The results of this analysis support this observation in Japanese patients, with all patients who completed 2 years of pembrolizumab with SD or better still being alive at the time of the data cutoff. The primary limitation of this study is the relatively small patient population and consequent small subgroup sizes of the various melanoma subtypes.

No new safety concerns were identified in this analysis. TRAEs occurring since the previous analysis included grade 1 or 2 urticaria (*n* = 4) and abdominal pain upper (*n* = 2), and grade 3 or 4 severe skin reaction (*n* = 1), type 1 diabetes (*n* = 1), and pemphigoid (*n* = 1).^9^ The immune‐mediated and infusion reactions reported were generally consistent with those of previous clinical trials evaluating pembrolizumab monotherapy in advanced melanoma.^12^ The general safety profile of pembrolizumab in this analysis was similar to that observed in other long‐term analyses of pembrolizumab in non‐Japanese patients.^7^, ^27^

In conclusion, the final analysis of KEYNOTE‐041 demonstrated that the long‐term safety of pembrolizumab monotherapy in Japanese patients with advanced melanoma was similar to the safety profile of pembrolizumab reported previously. Pembrolizumab continued to provide durable antitumor activity in Japanese patients. These findings provide further support for the use of pembrolizumab monotherapy in Japanese patients with advanced melanoma.

## CONFLICT OF INTEREST STATEMENT

KY, SF, HU (Uchi), TI, and HF have no conflicts of interest to declare. YF is an editorial board member of the *Journal of Dermatology* and a co‐author of this article. To minimize bias, they were excluded from all editorial decision‐making related to the acceptance of this article for publication. TT has received speakers’ bureau fees from Company Ono Pharmaceutical, MSD, Novartis Pharma, and Bristol‐Myers Squibb. YK has received honoraria of speakers’ fees from Company Ono Pharmaceutical Co., Ltd, Novartis Pharma K.K., AstraZeneca K.K., Otsuka Pharmaceutical Co., Ltd, Jansen Pharmaceutical K.K., Merck Biopharma Co., Ltd, Maruho Co., Ltd, and Bristol‐Myers Squibb K.K. KN have received honoraria of conference registration fees and/or travel or accommodation expenses from Company honoraria of speakers’ fees from Company Bristol‐Myers Squibb K.K. and Merck Biopharma Co., Ltd, and a followship, research grant or education grant from Company Ono Pharmaceutical Co., Ltd. HU (Uhara) has received honoraria and research funding from Company Ono Pharmaceutical Co., Ltd and Taiho Pharmaceutical. He has received honoraria from Company Novartis Pharma, Bristol‐Myers Squibb, and MSD. KN (Nakagawa) has received honoraria from Company Ono Pharmaceutical Co., Ltd.; Merck Biopharma Co., Ltd.; Amgen Inc.; Kyowa Kirin Co., Ltd.; Nippon Kayaku Co., Ltd.; Takeda Pharmaceutical Co., Ltd.; AstraZeneca K.K.; 3H Clinical Trial Inc; Chugai Pharmaceutical Co., Ltd.; Care Net, Inc; Eli Lilly Japan K.K.; Medical Review Co., Ltd.; MSD K.K.; Medical Mobile Communications co., Ltd/Pfizer Japan Inc.; YODOSHA CO., LTD.; Nippon Boehringer Ingelheim Co., Ltd.; Nikkei Business Publications, Inc.; Taiho Pharmaceutical Co., Ltd.; Japan Clinical Research Operations; Bayer Yakuhin, Ltd; CMIC Co., Ltd.; CMIC ShiftZero K.K.; Novartis Pharma K.K.; Life Technologies Japan Ltd.; TAIYO Pharma Co., Ltd.; Neo Communication; KYORIN Pharmaceutical Co., Ltd.; Roche Diagnostics K.K.; Bristol‐Myers Squibb K.K.; and AbbVie Inc. His institution has received research funding from Company PAREXEL International Corp.; Eisai Co., Ltd; PRA HEALTHSCIENCES; AstraZeneca K.K; EPS Corporation.; Mochida Pharmaceutical Co., Ltd.; Kissei Pharmaceutical Co., Ltd.; Covance Japan Inc.; EPS International Co., Ltd.; Japan Clinical Research Operations; Daiichi Sankyo Co., Ltd.; Takeda Pharmaceutical Co., Ltd.; Taiho Pharmaceutical Co., Ltd.; GlaxoSmithKline K.K.; MSD K.K.; Sanofi K.K.; Ono Pharmaceutical Co., Ltd; Chugai Pharmaceutical Co., Ltd.; PPD‐SNBL K.K; Nippon Boehringer Ingelheim Co., Ltd.; SymBio Pharmaceuticals Limited.; Sysmex Corporation; IQVIA Services JAPAN K.K.; Medical Research Support; SYNEOS HEALTH CLINICAL K.K; Eli Lilly Japan K.K.; Nippon Kayaku Co., Ltd.; Amgen Inc.; EP‐CRSU Co., Ltd./Novartis Pharma K.K.; Mebix, Inc.; Otsuka Pharmaceutical Co., Ltd.; Bristol‐Myers Squibb K.K.; SRL, Inc.; Janssen Pharmaceutical K.K.; Pfizer R&D Japan G.K.; AbbVie Inc.; A2 Healthcare Corp.; Bayer Yakuhin, Ltd; Pfizer Japan Inc., and Patents from Company Daiichi Sankyo Co., Ltd. He has received consulting or advisor role from Company Eli Lilly Japan K.K.; Ono Pharmaceutical Co., Ltd.; KYORIN Pharmaceutical Co., Ltd.; Pfizer Japan Inc. SH is employee of MSD K.K. Tokyo, Japan. MW is employee of MSD K.K. Tokyo, Japan and has stock option from Merck & Co., Inc., Rahway, NJ, USA. KN (Noguchi) is employee of MSD K.K. Tokyo, Japan and and has stock option from Merck & Co., Inc., Rahway, NJ, USA. NY has received advisory board from Company Merck and honoraria of speakers’ fee from Company Ono Pharmaceutical Co., Ltd, Bristol‐Myers Squibb, Novartis Pharma, and Amgen Inc.

## Supporting information


Table S1.

## Data Availability

Merck Sharp & Dohme LLC, a subsidiary of Merck & Co., Inc., Rahway, NJ, USA (MSD) is committed to providing qualified scientific researchers access to anonymized data and clinical study reports from the company's clinical trials for the purpose of conducting legitimate scientific research. MSD is also obligated to protect the rights and privacy of trial participants and, as such, has a procedure in place for evaluating and fulfilling requests for sharing company clinical trial data with qualified external scientific researchers. The MSD data sharing website (available at: http://engagezone.msd.com/ds_documentation.php) outlines the process and requirements for submitting a data request. Applications will be promptly assessed for completeness and policy compliance. Feasible requests will be reviewed by a committee of MSD subject matter experts to assess the scientific validity of the request and the qualifications of the requestors. In line with data privacy legislation, submitters of approved requests must enter into a standard data‐sharing agreement with MSD before data access is granted. Data will be made available for request after product approval in the United States and European Union or after product development is discontinued. There are circumstances that may prevent MSD from sharing requested data, including country or region‐specific regulations. If the request is declined, it will be communicated to the investigator. Access to genetic or exploratory biomarker data requires a detailed, hypothesis‐driven statistical analysis plan that is collaboratively developed by the requestor and MSD subject matter experts; after approval of the statistical analysis plan and execution of a data‐sharing agreement, MSD will either perform the proposed analyses and share the results with the requestor or will construct biomarker covariates and add them to a file with clinical data that is uploaded to an analysis portal so that the requestor can perform the proposed analyses.
