# Supplementary material for: Long‐term follow‐up results from KEYNOTE‐041: Phase 1b study of pembrolizumab in Japanese patients with advanced melanoma
Source: J Dermatol. 2024 Mar 26;51(5):632–42. doi: 10.1111/1346-8138.17002 (PMC11483956; doi:10.1111/1346-8138.17002)
Supplement: Supplementary file 1 — Table S1. [file JDE-51--s001.docx]

**Table S1. Summary of efficacy by melanoma subtype**

| Response evaluation | Lentigo maligna melanoma | Superficial spreading melanoma | Nodular melanoma | Acral lentiginous melanoma | Not classified | Total  Cutaneous | Mucosal |
| --- | --- | --- | --- | --- | --- | --- | --- |
| Patients in population | 1 | 3 | 9 | 12 | 4 | 29 | 8 |
| ORR, n (%, 95% CI)^a^ | 1  (100, 2.5-100.0) | 1  (33.3, 0.8-90.6) | 0  (NA) | 3  (25.0, 5.5-57.2) | 2  (50.0, 6.8-93.2) | 7  (24.1, 10.3-43.5) | 2  (25.0, 3.2-65.1) |
| Median PFS, months (95% CI)^a^ | NR  (NA) | 6.6  (4.2, NR) | 2.8  (2.6-7.0) | 4.1  (1.7-16.6) | 7.6  (2.8, NR) | 4.2  (2.8-7.0) | 3.4  (2.1-13.8) |
| Median OS, months (95% CI)^b^ | NR  (NA) | NR  (10.7, NR) | 18.4  (3.8, NR) | 25.1  (6.1, NR) | NR  (10.0, NR) | 25.1  (15.8, NR) | 8.7  (3.9, NR) |
| Median time to response, months (range)^a,c^ | 3.8  (4-4) | 2.7  (3-3) | NA  (NA) | 2.8  (3-4) | 2.9  (3-3) | 2.8  (3-4) | 4.1  (3-6) |
| Median response duration, months (range)^a,c,d^ | NR  (24+-24+) | 4  (4-4) | NA  (NA) | NR  (6-25+) | NR (10-25+) | NR  (4-25+) | NR  (8-20+) |

CI, confidence interval; NA, not applicable; NR, not reached; ORR, objective response rates; OS, overall survival; PFS, progression-free survival.

^a^Response and PFS was assessed per RECIST v1.1 by central review.

^b^Patients in population on OS analysis are all treated patients.

^c^Analysis on time to response and response duration are based on subjects with a best overall response as confirmed complete response or partial response only.

^d^From product-limit (Kaplan-Meier) method for censored data. The “+” symbol indicates there is no progressive disease by the time of last disease assessment.
